# PlMAPK10, a Mitogen-Activated Protein Kinase (MAPK) in *Peronophythora litchii*, Is Required for Mycelial Growth, Sporulation, Laccase Activity, and Plant Infection

**DOI:** 10.3389/fmicb.2018.00426

**Published:** 2018-03-08

**Authors:** Liqun Jiang, Junjian Situ, Yi Zhen Deng, Lang Wan, Dandan Xu, Yubin Chen, Pinggen Xi, Zide Jiang

**Affiliations:** ^1^Guangdong Province Key Laboratory of Microbial Signals and Disease Control, South China Agricultural University, Guangzhou, China; ^2^Guangdong Province Key Laboratory of New Technology in Rice Breeding, Rice Research Institute, Guangdong Academy of Agricultural Sciences, Guangzhou, China; ^3^Integrative Microbiology Research Centre, South China Agricultural University, Guangzhou, China

**Keywords:** *Peronophythora litchii*, mitogen-activated protein kinase, gene silencing, pathogenicity, laccase activity

## Abstract

Mitogen-activated protein kinase (MAPK) pathways are ubiquitous and evolutionarily conserved signal transduction modules directing cellular respond to a diverse array of stimuli, in the eukaryotic organisms. In this study, *PlMAPK10* was identified to encode a MAPK in *Peronophythora litchii*, the oomycete pathogen causing litchi downy blight disease. PlMAPK10, containing a specific and highly conserved dual phosphorylation lip sequence SEY (Serine-Glutamic-Tyrosine), represents a novel group of MAPKs as previously reported. Transcriptional profiling showed that *PlMAPK10* expression was up-regulated in zoospore and cyst stages. To elucidate its function, the *PlMAPK10* gene was silenced by stable transformation. *PlMAPK10* silence did not impair oospore production, sporangium germination, zoospore encyst, or cyst germination but hindered hyphal growth, sporulation, pathogenicity, likely due to altering laccase activity. Over all, our results indicated that a MAPK encoded by *PlMAPK10* gene in *P. litchii* is important for pathogenic development.

## Introduction

Litchi (*Litchi chinensis* Sonn.) is native to China with the hugest cultivated area in the world and production due to its lovely shape, beautiful color, delicious taste, and medicinal value (Jiang et al., 2006). However, litchi fruit does not reach maximum export volume from China, owing to suffering from serious plant diseases and insect pests, in which, litchi downy blight is the most serious disease (Wang et al., 2009). Litchi downy blight is caused by the oomycete pathogen *Peronophythora litchii*, with a broad range of infection stages and tissues including tender leaf, flower, and mature fruit period, and even extends to post-harvest and transport (Xu et al., 2013). Oomycetes are evolutionarily related to marine algae and has a far relationship from fungi although it grows filamentous mycelia and includes various of plant and animal pathogens responsible for many economically important diseases (Ye et al., 2016).

Mitogen-activated protein kinase (MAPK), with a conserved serine/threonine domain, is the center of cell signal transduction network. A variety of external or internal signals were shown to be transduced by MAPK pathway and regulate cell growth, development, stress response, and pathogenicity, etc. (Chen and Thorner, 2007). In the model fungus, *Saccharomyces cerevisiae*, five important cellular development events, namely sporulation, cell wall integrity, osmoregulation, sexual mating, and filamentous (pseudohyphal) growth, have been shown to be regulated respectively by five established MAPK pathways (Chen and Thorner, 2007; Vallejo and Mayinger, 2015). Specifically, *S. cerevisiae* Hog1 is integral to the osmoregulatory signal transduction cascade (Nancy et al., 2015), Fus3/Kss1 are essential for sexual reproduction and regulate chronological life span (Maneesha et al., 2017), and MPK1 is involved in cell wall integrity (Kim et al., 2018). In addition, a specialized type of MAPKs in fungi and mammalian was termed as stress-activated protein kinases (SAPKs), as it is responsible for stress response, including heat shock, hyperosmolarity, ultraviolet (UV) light irradiation, and oxidative stress (Brown et al., 2014). Examples of SAPKs from fungi include *Magnaporthe oryzae* YPD1 regulating penetration and conidiation (Mohanan et al., 2017), *Mycosphaerella graminicola* Hog1 involved in osmosensitivity and dimorphic transition in fungal pathogenicity (Mehrabi et al., 2006), and *Bipolaris oryzae* SRM1 for tolerance to hyperosmolarity, hydrogen peroxide (H_2_O_2_), and UV exposure (Moriwaki et al., 2006). On the other hand, mammalian p38s and JNKs are involved in the inflammatory and stress responses (Kyriakis et al., 2004; Chadee and Kyriakis, 2010). In the plant pathogenic oomycetes, three MAPKs/SAPKs in *Phytophthora sojae*, namely PsMPK1, PsMPK3 (PsSAK1) and PsMPK7, have been demonstrated essential for spore development, osmotic and oxidative stresses responses, and pathogenicity (Li et al., 2010, 2014; Gao et al., 2015).

In the long-term interaction between plant and microbial pathogens, plant has evolved defense responses, one of which is rapidly elevating level of reactive oxygen species (ROS) at the infection site (Park et al., 2018). As the first parclose for host plant against pathogens, ROS is further utilized by peroxidase and combines with polyphenols, such as callose deposition, lignin, and polymeric polyphenols synthesized (Torres and Dangl, 2005; de Leon and Montesano, 2017). In addition, ROS triggers the pathogen-associated molecular pattern-triggered immunity (PTI) by acting as a secondary messenger (Neill et al., 2002; Nürnberger et al., 2004; Zhang et al., 2009; Sang and Macho, 2017). Meanwhile, plant pathogens evolved ability to detoxify plant-derived ROS either by enzymatic (Staerck et al., 2017) or non-enzymatic mechanism, to ensure successful infection (Garg and Chandel, 2015).

In this study, we firstly functionally study *PlMAPK10*, a *P. litchii* ortholog to *P. sojae MAPK10* (Ye et al., 2015), by generation and characterization of *PlMAPK10*-silenced mutants, and examining the expression pattern during pathogenic development. Our results showed that *PlMAPK10* was highly expressed in infection-related structures, including zoospores and cysts, compared to vegetative mycelia. Silence of *PlMAPK10* gene led to reduced mycelial growth rate, reduced sporulation, weakened pathogenicity and altered laccase activity, indicating an important function of MAPK signal pathway in oomycete pathogenicity.

## Materials and Methods

### Bioinformatics Analysis

Genome sequence data of *P. litchii* were obtained from NCBI (BioProject ID: PRJNA290406) (Ye et al., 2016). JGI^1^ is used for prediction of *P. sojae* and *Phytophthora ramorum* genomic DNA and protein sequence. Genomic DNA and protein sequences of *Phytophthora infestans* were obtained from the Broad Institute^2^. The five STKc_MAPKs in the alignment of the indicated MAP kinases were predicted by SMART^3^ and CDD^4^ in NCBI. The phylogenetic dendrograms were constructed with Neighbor-Joining algorithm (with setting of 1000 bootstrap replications), by the MEGA 7.0 program^5^.

### *P. litchii* Strain and Culture Conditions

*Peronophythora litchii* strain was isolated from a litchi fruit with litchi downy blight disease in Guangdong province, and purified by single-spore isolation using MSM400 Dissection Microscope (Singer Instruments) (Jiang et al., 2017). The strain was cultured on carrot juice agar (CJA) media (juice from 200 g carrot topped up to 1 L, 15 g/L agar for solid media) at 25°C in the dark. The *P. litchii* transformants of *PlMAPK10*-silencing were maintained on CJA media containing 50 μg/L geneticin (G418) at 25°C in the dark and then transferred to be cultured on another CJA media plate without G418 at the same condition as wild type strain. Sporangia were harvested by flooding the mycelia, which had been cultured on CJA media for 5 days, with sterile distilled water, then filtering the subsequent suspension through a 100 μm mesh size of strainer. The suspension was incubated with sterile distilled water at 16°C for 0.5 and 2 h, respectively, for zoospores release. 10 μL sporangium suspension was sampled to calculate the release rate of zoospores under microscope.

### RNA Extraction and Gene Expression Analysis

Total RNA was extracted from different developmental stages and different time-points post-mycelial mat-inoculation on tender litchi leaves (Ye et al., 2011), using the Total RNA Kit (Omega, Cat. No.: R6834-01). RNA integrity was examined by agarose gel electrophoresis. For reverse transcription, 1 μg total RNA was used to synthesize the first-strand cDNA by oligo(dT) priming with the M-MLV reverse transcriptase kit (Invitrogen, Cat. No.: S28025-014). Transcription of *PlMAPK10* was analyzed with qRT-PCR assay using primers MAPK10-QRT-F: GCTCCACTTTAAGCCGAATG and MAPK10-QRT-R: ATTCGTCACCTTGCAGCTCT. *P. litchii actin* gene (using primers PlAct-F: TCACGCTATTGTTCGTCTGG and PlAct-R: TCATCTCCTGGTCGAAGTCC) was used as loading control and the relative fold change was calculated using the 2^-ΔΔCT^ method.

To analyze transcription of *PlMAPK10* gene in response to oxidative stress, WT hyphae were cultured at 25°C in the dark for 3 days using liquid carrot juice medium followed by washed with liquid Plich medium (0.5 g/L KH_2_PO_4_, 0.25 g/L MgSO_4_⋅7H_2_O, 1 g/L asparagine, 1 mg/L thiamine, 0.5 g/L yeast extract, 10 mg/L β-sitosterol, 5 g/L glucose, 15 g/L bacto-agar for solid media), and subsequently incubation in 20 mL liquid Plich medium (van West et al., 1999). After 0, 45, or 55 min respectively, appropriate H_2_O_2_ was added to the liquid Plich medium to reach a final concentration of 5 mM. At 60 min post-inoculation in liquid Plich medium, all samples were harvested and subject to examination of *PlMAPK10* expressional level, with a set of mycelial culture in liquid Plich medium for 60 min, untreated by H_2_O_2_, as control.

### Transformation of *P. litchii*

*PlMAPK10*-silenced mutants were generated by a polyethylene glycol (PEG)-mediated protoplast transformation strategy. The full-length open reading frame of *PlMAPK10* was amplified with primers MAPK10-FL-F: AAACGTACGATGACGAGCACGTCGTTG and MAPK10-FL-R:s AAAATCGATTTACTCGGCGATAGTCTCCTGT by PCR. The PCR fragment was ligated into the pTOR vector^6^ digested with *Cla*I and *BsiW*I in antisense orientation and sequenced for confirmation. 30 μg of the resulting construct pTOR::*PlMAPK10* was transformed, a selection construct carrying the geneticin gene *nptII*, into protoplasts of *P. litchii* wild type strain.

Preliminary transformants were identified according to their ability to grow on CJA media containing 50 μg/mL geneticin. Genome PCR analysis were then performed to confirm plasmid integration in these transformants, with primers ham34-F: GCTTTTGCGTCCTACCATCCG and MAPK10-F: AAACGTACGATGACGAGCACGTCGTTG. These transformants were then subjected to qRT-PCR assay for analysis of *PlMAPK10* expression level and gene-silencing efficiency.

### Pathogenicity Assays on Litchi Leaves

Pathogenicity on litchi was assayed using zoospore suspensions of *P. litchii* with two inoculation methods: (1) 10 μL zoospore suspension (10^4^ zoospores/L) was directly inoculated onto reverse side of a litchi tender leaf. Each strain was tested on 10 litchi leaves. The proportion of leaves displaying disease phenotype was calculated; (2) 30 mL sporangium suspension (10^4^ zoospores/L) was sprayed to three tender litchi branches, each of which contained approximately 30 leaves. According to the proportions of leaves with different disease levels, a disease severity value was calculated. Disease severity = [Σ (number of symptomatic leaves in certain index) × disease index]/[(total number of leaves investigated) × (the highest disease index)] × 100. The leaves were maintained at 80% humidity in the dark at 25°C. The symptoms were examined at 48 h post-inoculation. This experiment was repeated three times independently, with three biological replications per repeat.

### Extracellular Enzyme Activity Assays

Detection of peroxidase secretion was performed following the reported procedure (Sheng et al., 2015), based on laccase activity detection. At least three independent repeats, each with three biological replica, were performed for each instance.

### Identification, Selection, and Quantification of Putative Extracellular Laccase and Peroxidase Genes

We firstly searched the predicted extracellular laccase and peroxidase encoding genes in *P. litchii* genome according to their orthologs that annotated in the *P. sojae* genome and their conserved Cu-oxidase domains (IPR001117, IPR011706, and IPR011707). All the identified genes were quantified by qRT-PCR and analyzed by Multi-experiment Viewer program to select for those with significant fold change, during the life cycle in the wild type. All the selected genes were used to compare expression with wild type and CK strain by qRT-PCR. This experiment was repeated three independent times, and for each repeat with three biological replications.

### Hypersensitivity Toward Oxidative Stress

The sensitivity of *PlMAPK10*-silenced transformants to hydrogen peroxide (H_2_O_2_) was evaluated on modified Plich medium plates following established procedure (Sheng et al., 2015). At least three independent repeats, which contained three replicates, were performed for each instance. Growth inhibition rate was calculated, and the inhibition rate = (growth rate on plates without H_2_O_2_-growth rate on plates with H_2_O_2_)/growth rate on plates with H_2_O_2_.

## Results

### *PlMAPK10* Encodes a Typical and Conserved Mitogen-Activated Protein Kinase With a Dual Phosphorylation Lip

**FIGURE 1:**
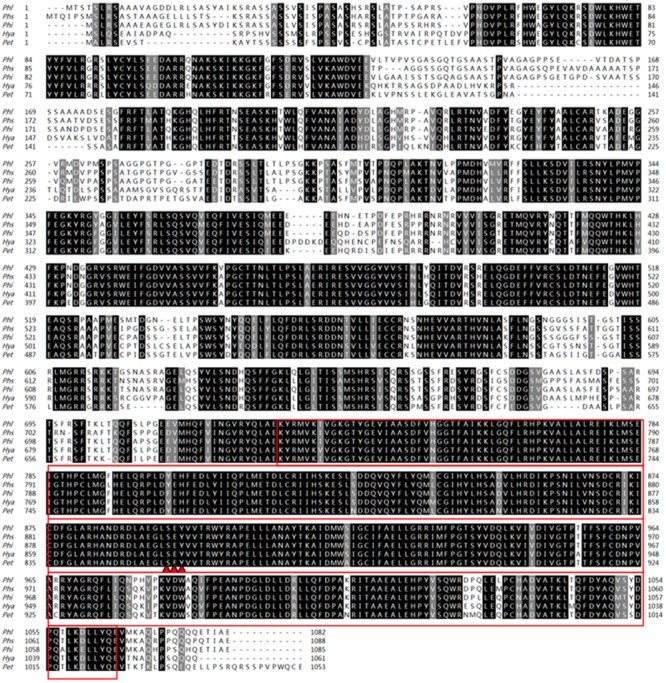
Amino acid sequences arrangement and phylogenetic analysis with PlMAPK10 protein and its orthologs. Amino acid sequences alignment of PlMAPK10 and its orthologs from *Phytophthora infestans* (Phi), *Phytophthora sojae* (Phs), *Hyaloperonospora arabidopsidis* (Hya), and *Peronospora tabacina* (Pet). The red boxes and three red triangles represent STKc_MAPK domains and predicted dual phosphorylation lip sequences, respectively.

The available genome data of *P. litchii* was searched against using full-length sequence of the *P. sojae* MAPK10 protein (PsMAPK10) through the program TBLASTN integrated in SeqHunter2.0 software (Ye et al., 2010), with an expected (E) value of 1.0E^-20^. A single gene, designated *PlMAPK10*, was identified in the search which encoded a protein of 85% amino acid identity with PsMAPK10 and the case spanning more than 90% of the length of the sequence. The corrected *PlMAPK10* gene model is 3249 bp in length with no intron, and encodes a protein of 1082 amino acids. We predicted a typically conservative serine/threonine protein kinase catalytic (STKc) MAPK domain (E = 8.14e-143; amino acids 748–1083) in PlMAPK10, using the CD-Search program on NCBI website. Sequence conservation between full-length PlMAPK10 protein and its orthologs in *P. sojae, P. infestans*, and *P. parasitica* is 85, 90, and 90%, respectively. We also noticed that a same predicted dual phosphorylation lip sequence SEY present in both *P. litchii* (amino acids 894–896) and several other oomycetes (**Figure 1**).

**FIGURE 2:**
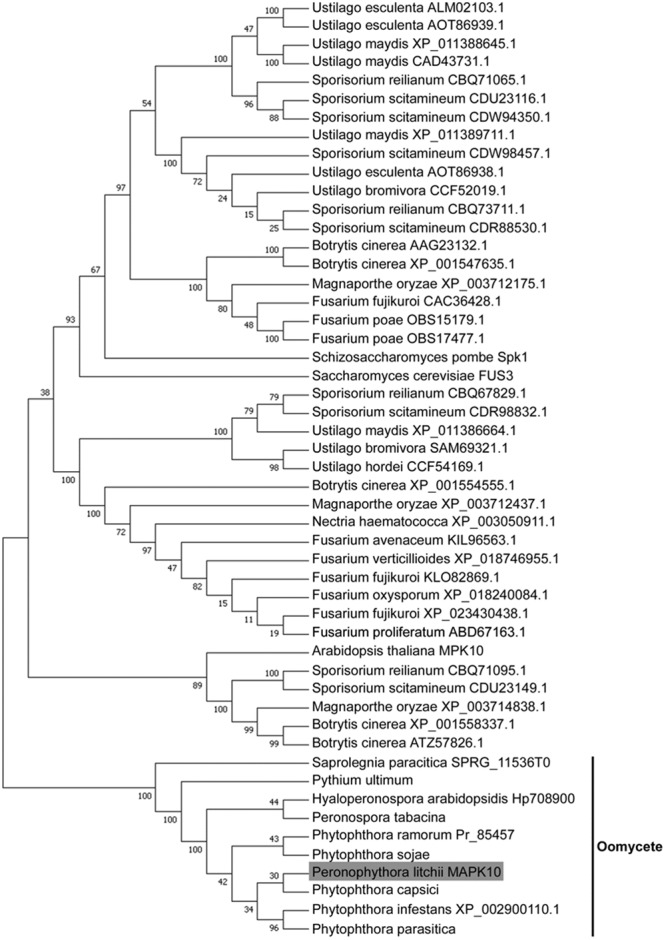
Phylogenetic analysis of MAPK10 proteins in *P. litchii* and in other oomycetes, pathogenic fungi, yeasts, and plant. Each sequence was labeled with its taxonomic name and accession number. The evolutionary history was inferred using the Neighbor-Joining method (Saitou and Nei, 1987). The optimal tree with the sum of branch length = 4.63768252 is shown. The evolutionary distances were computed using the JTT matrix-based method (Jones et al., 1992) and are in the units of the number of amino acid substitutions per site. The analysis involved 51 amino acid sequences. All positions with less than 50% site coverage were eliminated. That is, fewer than 50% alignment gaps, missing data, and ambiguous bases were allowed at any position. There were a total of 371 positions in the final dataset. Evolutionary analyses were conducted in MEGA7 (Kumar et al., 2016).

Besides the 10 oomycete MAPKs, we search the orthologous sequence of PlMAPK10 by BLASTP using its full length sequence, in the pathogenic fungi including genus of *Ustilago* (taxid: 5269), *Sporisorium* (taxid: 63265), *Fusarium* (taxid: 5506), *Botrytis* (taxid: 33196), and in the rice-blast fungus *Magnaporthe oryzae* 70–15 (taxid: 242507). The top 3–10 hits (>40% amino acid identity, E value ≤ 1.0E^-20^) from each search were retrieved for sequence alignment and phylogenetic analysis. We also searched the similar MAPKs from plant *Arabidopsis thaliana*, and from the yeasts *S. cerevisiae* and *Schizosaccharomyces pombe* by BLink, and included them for phylogenetic analysis. The phylogenetic analysis showed that the 10 oomycete MAPK10 orthologs were in a clade that seperated from the other kinases (**Figure 2**), and they were mostly conserved in the serine/threonine protein kinase catalytic (STKc) MAPK domain. Therefore we conclude that PlMAPK10 was conserved with its orthologs of the indicated two *Phytophthora* species and three downy mildew species in full length protein sequence.

### Generation of *PlMAPK10*-Silenced Transformants

**FIGURE 3:**
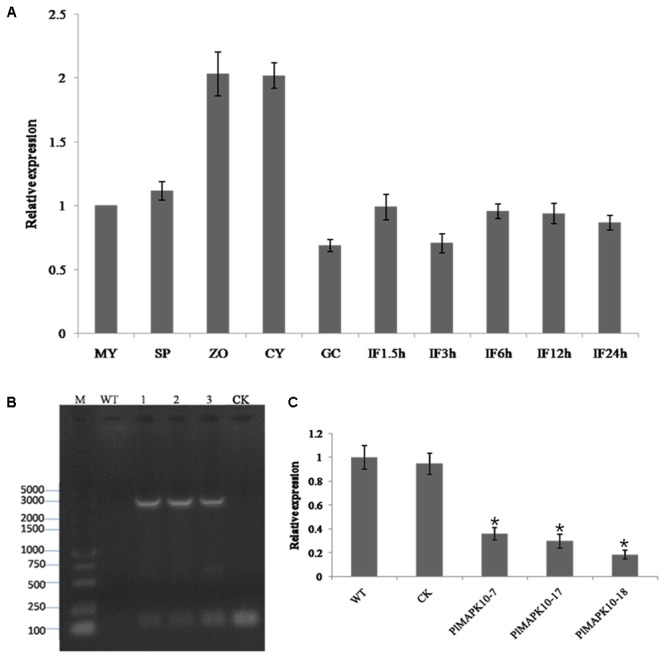
Transcriptional profile of *PlMAPK10* gene in the life cycle and infection stages, and identification and selection of *PlMAPK10*-silenced transformants. **(A)** Relative expression levels of *PlMAPK10* were determined by qRT-PCR with total RNA extracted from specific-stages of life cycle: MY, mycelia; SP, sporangia; ZO, zoospores; CY, cysts; GC, germinating cysts. IF1.5h–IF24h, infection stage of 1.5, 3, 6, 12, and 24 h post-inoculation, using mycelial mats on the tender litchi leaves. Expression levels were normalized using the MY values as ‘1.’ *P. litchii Actin* gene was used as endogenous gene. The experiment was repeated two times with independent sampling. **(B)** Genome PCR identification of three transformants, CK strain and WT strain. Marker: DL 5000. Lane 1, lane 2, and lane 3 were the three *PlMAPK10*-silenced transformants, named *PlMAPK10*-7, *PlMAPK10*-17, and *PlMAPK10*-18, respectively. **(C)** Relative gene expression levels of *PlMAPK10* in *PlMAPK10*-7, *PlMAPK10*-17, and *PlMAPK10*-18 and the WT and CK strains. *PlMAPK10* expression was normalized to that of WT value (set as “1.0”). *P. litchii Actin* gene serves as a loading control. Asterisks represent significant difference (*P* < 0.05) based on statistics analysis using SPSS (version 19.0). These experiments were repeated three times independent, and for each repeat with three biological replications.

Next we examine the transcriptional patterns of *PlMAPK10* in mycelia, sporangia, zoospores, cysts, germination cysts and infection stages, by quantitative real-time PCR (qRT-PCR). *PlMAPK10* Transcripts in zoospores and cysts were estimated as twofold of that in mycelium, while not obviously changed in sporangia, germination cysts and during the infection process (**Figure 3A**). Thus, we infer that *PlMAPK10* may play a crucial role in zoospores and cysts.

To explore the biological functions of *PlMAPK10* in *P. litchii* development, stress response, and pathogenicity, we generated an antisense direction of PlMAPK10 coding region, driven by the oomycete constitutive pHAM34 promoter in a pTOR vector (Whisson et al., 2007) and transformed it into WT *P. litchii* protoplast cells following a well-established protocol (Jiang et al., 2017). Transformation with pTOR vector served as blank control and named as CK strain. In total, we obtained 132 transformants by antibiotic resistance screening. Among them, 49 transformants with stable integration of silence cassette into *P. litchii* genome, were identified by genome PCR amplification (**Figure 3B**). Finally, using qRT-PCR we identified 3 (T7, T17, and T18), out of 49 *PlMAPK10*-integrated transformants, showing significant (*P* < 0.05) lower transcriptional level of *PlMAPK10* than that in the wild type or CK control (**Figure 3C**). In the following we characterized mycelium growth, sporulation, and zoospore release of these three *PlMAPK10*-silenced strains and compared to WT and CK strains.

### *PlMAPK10*-Silenced Transformants Showed Slower Mycelium Growth and Less Sporulation But No Influence in Zoospore Release

**FIGURE 4:**
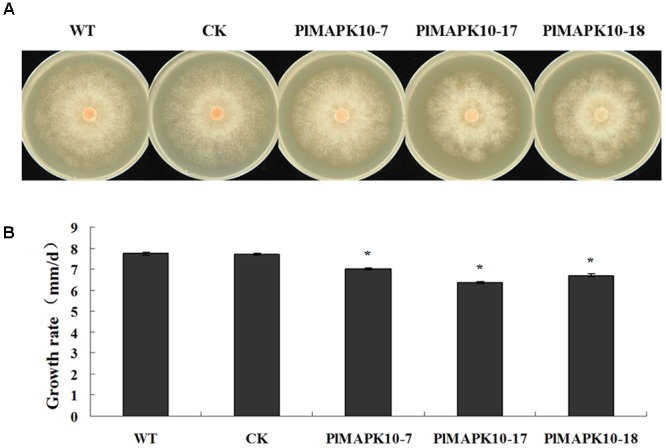
Measurement of colony diameter of WT, CK, and the *PlMAPK1*0-silenced transformants, *PlMAPK10*-7, *PlMAPK10*-17, and *PlMAPK10*-18. **(A)** Photographs of colony of WT, CK, and the *PlMAPK1*0-silenced transformants, *PlMAPK10*-7, *PlMAPK10*-17 and *PlMAPK10***-**18, were taken at 7 days. **(B)** Measurement of colony growth rate of WT, CK, and the three *PlMAPK1*0-silenced transformants. With “^∗^” on the top of each bar represents significant difference between *PlMAPK10*-silenced transformants and two controls based on the statistics analysis of Student’s *t*-test (^∗^*P* < 0.05). This experiment was repeated three times independent, and for each repeat with three biological replications.

We first measured colony diameters of the three silenced transformants as well as WT and CK strains. As shown in **Figure 4A**, the colony size of the *PlMAPK10*-silenced strains was obviously smaller than that of WT or CK strains. We further calculated the mycelial radial growth rate, and found that the rates of three *PlMAPK10*-silenced transformants were ranged from 6.36 to 7.00 mm/d, and were significantly (*P* < 0.05) lower than that of wild type (7.74 mm/d) and CK strains (7.71 mm/d) (**Figure 4B**).

**FIGURE 5:**
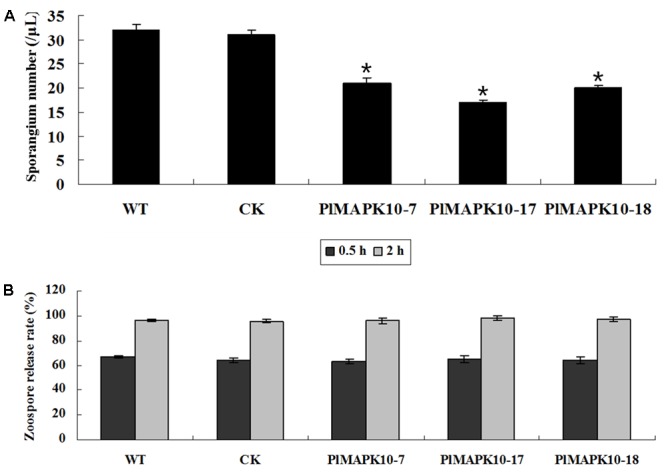
Measurement of sporangia number and zoospores release of *PlMAPK10*-silenced transformants, wild type and CK strain. **(A)** Mean ± SE sporangia number in 1 μl sporangia suspension. **(B)** Mean ± SE zoospore release rate. With “^∗^” on the top of each bar represents significant difference between *PlMAPK10*-silenced transformants and two controls (*P* < 0.05), based on the statistics analysis using SPSS (version 19.0) with statistical analysis of two-tailed *t*-test. This experiment was repeated three times independent, and for each repeat with three biological replications.

Next we assessed production of sporangia in the silenced strains. The average numbers in per μL sporangium suspension of the three *PlMAPK10*-silenced transformants were 21 in *PlMAPK10*-7, 17 in *PlMAPK10*-17, and 20 in *PlMAPK10*-18 respectively, significantly (*P* < 0.05) less than 32 in wild type and 31 in CK control (**Figure 5A**). However, zoospore release from an equal number of sporangia was not affected by *PlMAPK10* silencing. Zoospore release was induced in sporangium suspensions of three silenced transformants and two controls, by incubation at 16°C for 0.5 and 2 h, respectively. As shown in **Figure 5B**, after 0.5 h of incubation, an average of 63.15, 64.76, and 64.10% of the sporangia of *PlMAPK10*-7, *PlMAPK10*-17, and *PlMAPK10*-18, respectively, released zoospores, which was comparable to that in the controls (66.72% for WT and 64.16% for CK strain). After 2 h of incubation, an average of 95.77, 98.12, and 96.94% of the sporangia of *PlMAPK10*-7, *PlMAPK10*-17, and *PlMAPK10*-18, respectively, released zoospores, compared with 95.96 and 95.59% in the controls (WT and CK, respectively).

### *PlMAPK10* Is Required for Pathogenicity

**FIGURE 6:**
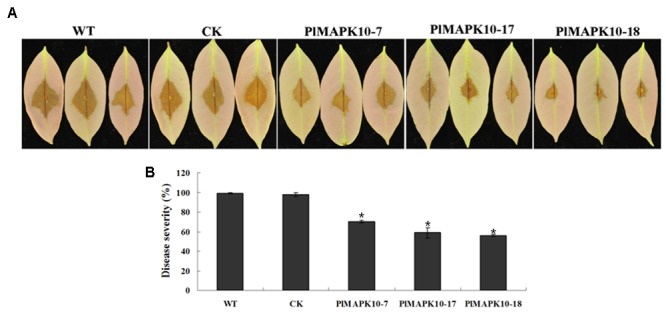
Pathogenicity test of *Peronophythora litchii PlMAPK10*-silenced transformants, WT and CK. **(A)** Zoospore suspension was inoculated on tender litchi leaves (*n* = 30 for each strain) for 48 h at 25°C in the dark. Images are three representatives for each instance. **(B)** Quantification of pathogenicity by disease severity values. Zoospore suspension was sprayed to tender litchi branches (containing approximately 30 leaves), for three independent repeats. With “^∗^” on the top of each bar represents significant difference between *PlMAPK10*-silenced transformants and two controls (*P* < 0.05), based on the statistics analysis using SPSS (version 19.0) with statistical analysis of two-tailed *t*-test. This experiment was repeated three times independent, and for each repeat with three biological replications.

To explore the roles of *PlMAPK10* in *P. litchii* pathogenicity we performed infection assay with tender litchi leave. Sporangium and zoospore suspensions of the three silenced transformants and the two control strains were respectively inoculated onto the leave surface and leave vein. For leave surface infection, zoospore suspensions were inoculated on the reverse side of tender litchi leaves and symptoms were examined on 2 days post-inoculation (dpi). We observed that the leaves inoculated with control zoospores showed typical disease symptoms and water-soaked lesions at 2 dpi (**Figure 6A**), while the lesion caused by the three *PlMAPK10*-silenced transformants was smaller (**Figure 6A**). To mimic a more realistic pathogenic process, sporangia suspensions were sprayed to tender litchi branches. According to the proportions of leaves of different disease levels, a disease severity value was calculated (see section “Materials and Methods”). As shown in **Figure 6B**, after 2 days of inoculation, an average disease severity value of 70.19, 58.89, and 56.30 of *PlMAPK10*-7, *PlMAPK10*-17, and *PlMAPK10*-18 sporangia, respectively, significantly (*P* < 0.05) lower when compared with 98.89 and 97.78 in the controls (WT and CK, respectively). Therefore, *PlMAPK10* might be critical for full pathogenicity of *P. litchii*.

### Silencing of *PlMAPK10* Decreases Extracellular Laccase Activity Rather Than Peroxidase Activity Through Regulating the Transcription of Relative Gene

**FIGURE 7:**
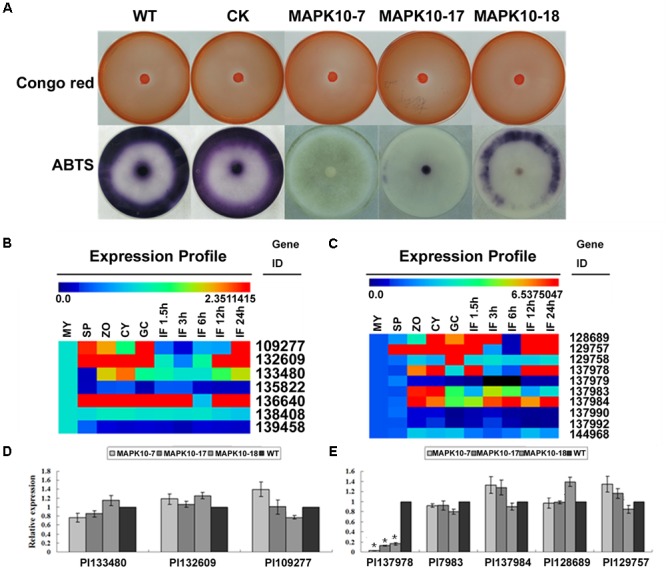
Detection of extracellular peroxidase and laccase activity, transcriptional patterns and relative expression of their putative genes in the *PlMAPK10*-silenced transformants. **(A)** Upper panel: peroxidase activity assay. Mycelial mats of WT, CK, and three *PlMAPK10*-silenced transformants were inoculated on solid Plich medium containing Congo Red at a final concentration of 500 μg/ml, as indicator. The strains were incubated for 24 h before Congo Red discoloration was assessed. Lower panel: laccase activity assay. Mycelial mats of the aforementioned strains were inoculated on LBA media containing 0.2 mM ABTS for 10 days, and the oxidized ABTS (dark purple) was measured as an index of laccase activity. **(B,C)** Transcriptional levels of selected peroxidase-encoding genes and laccase-encoding genes were respectively treated with Multi-experiment Viewer according to the genome of *P. litchii* and homology genes in *P. sojae* and their conserved domains. **(D,E)** Quantitative real-time PCR analysis of *P. litchii* putative peroxidase-encoding genes and laccase-encoding genes in three *PlMAPK10*-silenced transformants and wild type strain, respectively. This experiment was repeated three times independent, and for each repeat with three biological replications. Asterisks represent significant difference (*p* < 0.05) based on statistics analysis using SPSS (version 19.0).

Extracellular laccase or peroxidase activity has been shown to contribute to fungal or oomycete pathogenicity, therefore we further investigate any alteration in such extracellular enzymes activities in the *PlMAPK10*-silenced transformants, which may account for their reduced pathogenicity. We first assessed peroxidase activity based on Congo Red (CR) degradation. As shown in **Figure 7A** (Upper panel), diameters of the halo caused by the three *PlMAPK10*-silenced transformants were comparable to that of controls. Subsequently, we examined laccase activity based on oxidation assay of ABTS [2,2′-azino-*bis* (3-ethylbenzothiazoline-6 sulfonic acid)]. Three *PlMAPK10*-silenced transformants showed significantly decreased accumulation of ABTS, visualized by dark purple staining around the mycelial mat compared with wild type and CK control (**Figure 7A**, Lower panel). These results indicated that *PlMAPK10* may regulate extracellular laccase activity to ensure successful host infection.

To further find out the reason for the reduced laccase activity, we examined the transcription of putative peroxidase or laccase encoding genes. These genes were selected from a thorough search in *P. litchii* genome, using their orthologs annotated in the *P. sojae* genome and their conserved domains. As shown in **Figures 7B,C**, seven predicted peroxidase encoding genes (Pl_109277, Pl_132609, Pl_133480, Pl_135822, Pl_136640, Pl_138408, Pl_139458) and 10 predicted laccase encoding genes (Pl_128689, Pl_129757, Pl_129758, Pl_137978, Pl_137979, Pl_137983, Pl_137984, Pl_137990, Pl_137992, Pl_144968) existed in *P. litchii* genome. Among them, three of putative peroxidase genes (Pl_109277, Pl_132609, Pl_133480) and five of laccase genes (Pl_128689, Pl_129757, Pl_137978, Pl_137983, Pl_137984) were indicated to express differentially during infection stages based on Multi-experiment Viewer program analysis^7^, and were selected for further confirmation by qRT-PCR. Compared with wild type and CK, the transcript levels of three predicted peroxidase genes were not obviously changed in three *PlMAPK10*-silenced transformants (**Figure 7D**). In contrast, one laccase encoding gene (Pl_137978) out of the five predicted laccase genes, showed significant decrease in transcript level in three *PlMAPK10*-silenced transformants (73–97%) compared to that of WT and CK (**Figure 7E**). Since silencing of *PlMAPK10* affects the transcript level of a predicted laccase gene, we infer that PlMAPK10 act as a regulator in *P. litchii* pathogenicity through direct or indirect regulation on the transcription of laccase gene expression, likely serving a function in detoxification of raised ROS level caused by plant defense response.

### Expression of *PlMAPK10* Gene Is Not Regulated by Hydrogen Peroxide, While Silencing of *PlMAPK10* Has No Effect on Sensitivity to H_2_O_2_

Given that *PlMAPK10* is required for transcriptional induction of laccase gene Pl_137978, we would like to investigate whether *PlMAPK10* is essential for oxidative stress response. A sensitivity assay was performed with the three silenced strains and two control strains. The three *PlMAPK10*-silenced transformants displayed comparable H_2_O_2_ sensitivity with that of the control strains, as the diameters of all the examined strains were similar (Supplementary Figure S1A). As shown in Supplementary Figure S1B, inhibition rate of three *PlMAPK10*-silenced transformants and wild type were approximate in both 2 mM H_2_O_2_ and 5 mM H_2_O_2_. Based on this, we further detected the relative expression of *PlMAPK10* gene in mycelia treated with 5 mM H_2_O_2_ for different time. qRT-PCR assay showed that transcription of *PlMAPK10* gene did not change with H_2_O_2_ for different time (Supplementary Figure S1C). Thus, we inferred that expression of *PlMAPK10* gene is not induced by oxidative stress.

## Discussion

Mitogen-activated protein kinase is protein kinase highly conserved in eukaryotic organisms as signal transducers (Román et al., 2007). In this study, we identified a *P. litchii* ortholog of *MAPK10* gene, that is highly conserved in sequenced *Phytophthora* spp. and in the *Hyaloperonospora arabidopsidis* genome. Particularly, a typical STKc_MAPKs domains with the dual phosphorylation lip sequence SEY is present in all the oomycete MAPK10s.

The transcription of *PlMAPK10* gene was constitutive throughout the *P. litchii* life cycle, while up-regulated specifically in the zoospores and cysts. Given that signal transduction through MAPK pathways depends on a protein phosphorylation/dephosphorylation cascade, we could not ruled out that *PlMAPK10* may also play a role in infection process, transcriptional levels did not changed obviously in the time-course examination. Silencing of *PlMAPK10* expression in *P. litchii* reduced mycelial growth rate and sporangium sporulation, and without difference on zoospore release. *PlMAPK10*-silenced transformants lost pathogenicity to tender litchi leaves, which is consistant with the well-established function of MAPKs fungal development and pathogenesis (Xu, 2000; Zhao et al., 2005; Román et al., 2007). For example, knocking out *PMK1* and *CHK1* in *Magnaporthe grisea* and *Cochliobolus heterostrophus*, affects on appressoria and conidia formation respectively (Zhao et al., 2005; Eliahu et al., 2007). FMK1 in *Fusarium oxysporum*, GPMK1 in *Fusarium graminearum*, BMP1 in *Botrytis cinerea*, BMK1 in *Bipolaris oryzae*, and MgFUS3 in *Mycosphaerella graminicola* were demonstrated to be essential for pathogenicity (Zheng et al., 2000; Jenczmionka et al., 2003; Moriwaki et al., 2006; Kramer et al., 2009; Rispail and Di, 2009). In addition, deletion of *Botrytis cinerea SAK1* resulted in interruptived conidia formation, increased sclerotial development, and lost penetration to unwounded plant tissue (Segmüller et al., 2007), and knocking out of *Mycosphaerella graminicola Hog1* also affects on filamentous growth and pathogenicity (Mehrabi et al., 2006). Although functionally conserved, the amino acid sequences of oomycete MAPK10s were distance from a variety of fungal MAPKs, providing an addition evidence for the independent evolutionary lineage between phytopathogenic oomycetes and fungi.

During a long term of plant–microbe interaction, plants evolve a series of defense responses to protect themselves from the pathogens. During the process of coevolution with their hosts, pathogenic fungi or oomycetes developed a set of mechanisms to escape or overcome host plant cellular environment, the first parclose (Kim et al., 2009). We showed that the extracellular laccase activity of *PlMAPK10*-silenced transformants were much lower, and one putative laccase encoding gene was down-regulated due to the silence of *PlMAPK10* gene, suggesting a possible reason why the *PlMAPK10* is required for full pathogenicity. Silencing of *PsMPK7* in *P. sojae* leads to a similar result of reduced pathogenicity and extracellular laccase activity in the transformants compared with wild type and CK strains (Gao et al., 2015). Given that laccase or peroxidase has been widely identified in fungi as an important virulence factor (Zhu and Williamson, 2004; Molina and Kahmann, 2007; Howlett et al., 2009; Staerck et al., 2017), we infer that reduced pathogenicity of *PlMAPK10*-silenced transformants may be due to a severely compromise in degradation of callose deposition, lignin accumulation, and/or other stress tolerance capabilities during infection. However, it remains unclear whether *PlMAPK10* regulates the secretion pathway of extracellular enzymes.

Overall, our research suggests that PlMAPK10 is required for oomycete pathogenicity, likely via regulation of expression of gene encoding extracellular enzymes, which is consistant with several plant pathogenic fungi as reported. However, the regulatory mechanisms related to development and pathogenicity of *P. litchi*i awaits elucidation. The functional characterization of *P. litchii* MAPK10 will add to our knowledge of oomycete morphogenesis, pathgenesis, extracellular enzymes synthesis and secretion pathway, and provide a potential target of disease control.

## Author Contributions

This study was conceived and designed by ZJ, PX, and LJ. All experiments in this study were performed by LJ, JS, LW, and YC. Data was analyzed by LJ and DX. The guidance for this study was provided by ZJ, PX, and YD. This manuscript was written by LJ, ZJ, PX, and YD. All authors contributed to manuscript revision, read and approved the submitted version.

## Conflict of Interest Statement

The authors declare that the research was conducted in the absence of any commercial or financial relationships that could be construed as a potential conflict of interest.

## Footnotes

1 http://www.jgi.doe.gov/

2 http://www.broad.mit.edu/

3 http://smart.embl-heidelberg.de

4 https://www.ncbi.nlm.nih.gov/cdd/

5 http://megasoftware.net

6 https://www.ncbi.nlm.nih.gov/nuccore/EU257520.1

7 http://www.tm4.org
