# Supplementary material for: PlMAPK10, a Mitogen-Activated Protein Kinase (MAPK) in Peronophythora litchii, Is Required for Mycelial Growth, Sporulation, Laccase Activity, and Plant Infection
Source: Front Microbiol. 2018 Mar 8;9:426. doi: 10.3389/fmicb.2018.00426 (PMC5852060; doi:10.3389/fmicb.2018.00426)
Supplement: Supplementary file 1 [file Image_1.PDF]

Supplementary Material

PlMAPK10, a mitogen-activated protein kinase (MAPK) in *Peronophythora litchii*, is required for mycelial growth, sporulation, laccase activity and plant infection

Liqun Jiang, Junjian Situ, Yi Zhen Deng, Lang Wan, Dandan Xu, Yubin Chen, Pinggen Xi*, Zide Jiang*

***Correspondences:** Zide Jiang, zdjiang@scau.edu.cn; Pinggen Xi, xpg@scau.edu.cn

Supplementary Figure

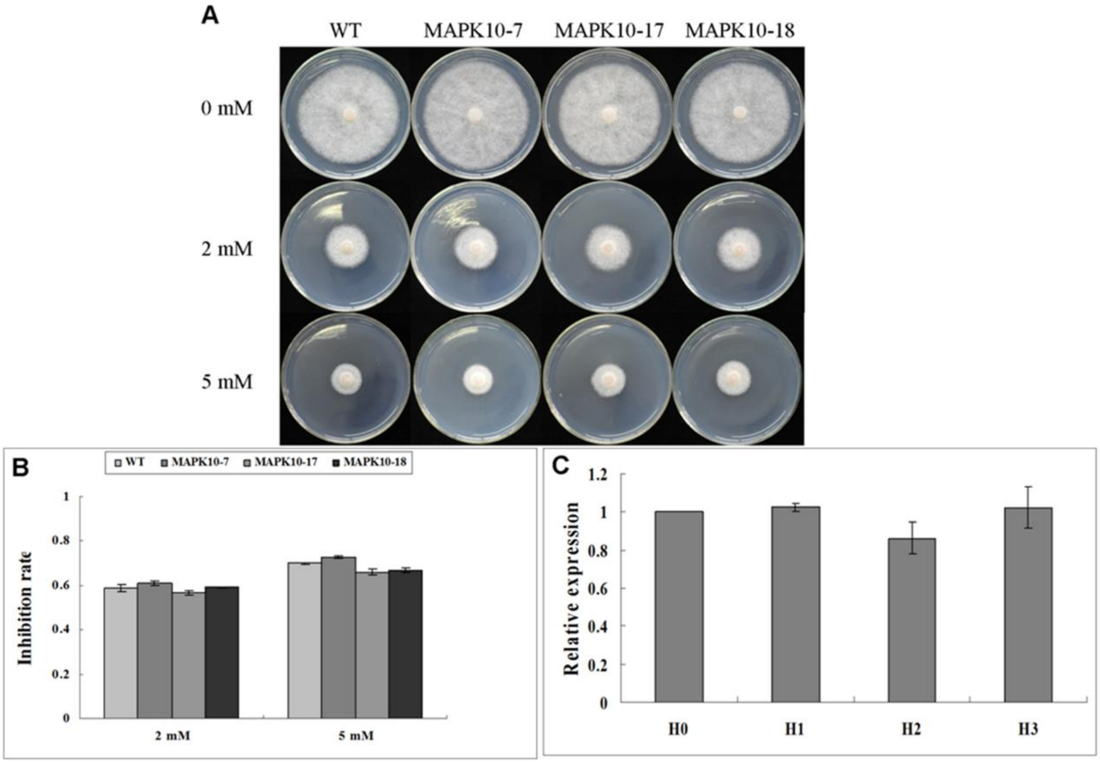

Supplementary Figure S1. Hypersensitivity test and relative expression level in *PlMAPK10*-silenced transformants. (A) Analysis of sencitivity to H_2O_2 with the WT stain and three *PlMAPK10*-silenced transformants. (B) Inhibition rates of the WT stain and *PlMAPK10*-silenced transformants were calculated based on their growth rate. (C) Transcriptional analysis of *PlMAPK10* gene under oxidative stress. H0, H1, H2 and H3 represent mycelia of wild type treated with 5 mM H_2O_2 for 0, 5, 15 and 60 min respectively. Same letter on the top of each bar represents that difference is not significant, while different letters for significant difference, based on statistics analysis using SPSS (version 19.0) with statistical analysis based on two-tailed t-test ($P < 0.05$).

Disclaimer: Although utmost care has been taken to ensure the correctness of the caption, the caption text is provided "as is" without any warranty of any kind. Authors advise the user to carefully check

the caption prior to its use for any purpose and report any errors or problems to the authors immediately (www.megasoftware.net). In no event shall the authors and their employers be liable for any damages, including but not limited to special, consequential, or other damages. Authors specifically disclaim all other warranties expressed or implied, including but not limited to the determination of suitability of this caption text for a specific purpose, use, or application.
